# Hybrid Carbonyl Iron/Iron Oxide Microfiber Textile Membranes with Magnetically Tunable Capacitance Under Compressive Loading

**DOI:** 10.3390/mi17040478

**Published:** 2026-04-15

**Authors:** Ioan Bica, Eugen Mircea Anitas, Octavian Madalin Bunoiu, Liviu Chirigiu, Gabriel Pascu

**Affiliations:** 1 Department of Physics, West University of Timisoara, V. Parvan Avenue 4, 300223 Timisoara, Romania; ioan.bica@e-uvt.ro (I.B.); madalin.bunoiu@e-uvt.ro (O.M.B.); gabriel.pascu@e-uvt.ro (G.P.); 2 Department of Physics, Craiova University, A. I. Cuza Street 13, 200585 Craiova, Romania; 3 Joint Institute for Nuclear Research, Joliot-Curie 6, 141980 Dubna, Russia; 4 Horia Hulubei, National Institute of Physics and Nuclear Engineering, 077125 Bucharest-Magurele, Romania; 5 Faculty of Pharmacy, University of Medicine and Pharmacy Craiova, Petru Rares 2, 200349 Craiova, Romania; liviuchirigiu@gmail.com

**Keywords:** magnetodielectric effect, magnetorheological suspension, microwave microplasma, iron oxide microfibers, carbonyl iron, cotton textile composite, planar capacitor

## Abstract

Flexible textile membranes were prepared by impregnating woven cotton fabrics with silicone oil (SO)-based suspensions containing carbonyl iron (CI) microparticles and iron oxide microfibers (μFe). The microfibers were obtained by a microwave-assisted microplasma process and then co-dispersed with CI in SO. In the final membranes, the CI content was kept constant at $\Phi_{\mathrm{CI}}=10$ vol.%, whereas the microfiber fraction was 0, 10 and 20 vol.%. The resulting membranes were used as dielectric layers in planar capacitors and examined at 1 kHz under a static magnetic field of up to 150 mT and compressive pressure up to 10 kPa. In every composition, the capacitance rose with increasing magnetic flux density, but both the zero-field capacitance and the field-induced capacitance change became smaller as the microfiber content increased. A monotonic, nearly linear increase in capacitance was also observed under compression over the tested pressure range. Within a simplified parallel-plate and magnetic-stress analysis, the capacitance data were further used to estimate the apparent relative permittivity, together with capacitance-derived indicators of deformation and stiffness. These estimates suggest field-induced stiffening of the membranes and a higher apparent low-field stiffness at higher microfiber loading. The obtained hybrid CI/μFe-based textile membranes can serve as composition-tunable dielectric layers whose electrical response is influenced by both magnetic field and compressive loading, making them relevant for flexible capacitor-based elements.

## 1. Introduction

Flexible capacitive sensors are widely used in wearable systems, human–machine interfaces, and robotic sensing because they combine mechanical flexibility, conformability, and low-power operation [1,2,3]. In the parallel-plate description, the capacitance depends on the electrode area, the electrode spacing, and the apparent relative permittivity $\varepsilon_{r}$ of the dielectric layer. Mechanical deformation generates an electrical signal by changing one or more of these parameters [1,4]. Therefore, the structure, stiffness, and dielectric response of the dielectric layer largely determine how the sensor deforms under load, and thus its sensitivity and operating range [1,4,5]. This has stimulated interest in dielectrics whose response can be tuned by mechanical loading, together with additional external stimuli.

Magnetically responsive composites provide one such route. In magnetoactive elastomers and magnetorheological suspensions (MRSs), magnetizable microparticles are dispersed in an electrically insulating matrix [6,7]. Under a static magnetic field, the particles become magnetized and interact through dipolar forces, promoting chain-like or columnar aggregates aligned with the field [8,9]. This restructuring modifies the internal microgeometry and the local electric-field distribution and is commonly accompanied by a pronounced change in the apparent dielectric response [6,9,10]. Carbonyl iron (CI) microparticles are among the most widely used soft-magnetic fillers in such systems [8,9,11,12]. CI consists of nearly spherical soft-magnetic iron microparticles and is especially attractive as a primary field-responsive filler because of its strong magnetic response and high saturation magnetization, which are generally greater than those of many oxidized iron compounds.

However, the use of spherical CI microparticles alone can limit the development of lightweight soft dielectrics because their high density tends to reduce sedimentation stability [13]. One way to expand tunability is to introduce a fibrous magnetic phase that restructures the field-responsive architecture without requiring very high filler loadings. A promising complementary phase is provided by iron oxide microfibers (μFe), which have substantially lower density and lower saturation magnetization than CI [14]. Here, the μFe component acts mainly as a secondary structural phase that can modify the spacing and arrangement of CI-rich domains while contributing to the structural reinforcement of the composite. Compared with iron oxide nanoparticles, a microfiber morphology is advantageous because its higher aspect ratio and network-forming character can influence the field-responsive architecture effectively, even at relatively low loading [15,16]. Such a combination of a strongly magnetic particulate phase and a lighter fibrous oxide phase is particularly attractive for hybrid field-responsive dielectrics.

Hybrid textile-like composites that combine cotton fibers, CI microparticles, and the μFe-derived iron oxide phase are particularly interesting in this respect. In such systems, the microfiber component can reduce the effective density and act as an anti-sedimentation medium, while the dielectric response varies systematically with microfiber fraction and magnetic flux density (*B*) [17]. These behaviors arise from microfiber-induced changes in the spacing and arrangement of CI magnetic dipoles [17]. Microfiber concentration controls both magneto-dielectric and viscoelastic characteristics, and fiber-thread scaffolds can support particle chains and improve shear or damping under field conditions [18,19]. Such results motivate hybrid particle–fiber dielectrics in which the magnetocapacitive response can be tuned through magnetic loading and phase morphology [15,17]. These considerations make textile-based hybrid particle–fiber dielectrics particularly attractive for flexible capacitor structures.

Textile dielectrics are attractive for wearable capacitive devices because they can undergo large reversible thickness changes while maintaining flexibility and, in some cases, high air permeability [20,21]. Natural fibers such as cotton and cellulose strengthen this approach by offering abundant, skin-friendly scaffolds that can be electrically functionalized. Cellulosic materials are widely used in wearable sensors [22], and cotton fabrics have been modified with conductive electrodes and dielectric additives to improve capacitive performance [23]. However, these textile sensors predominantly rely on mechanically induced capacitance changes, whereas the integration of magnetodielectric tunability into porous textile dielectrics remains comparatively underexplored.

The purpose of the present research is to determine how the μFe content tunes the device-level capacitance response of CI/μFe textile membranes under magnetic field and compressive loading, and to clarify the associated apparent dielectric and mechanical trends. The main novelty of this work lies in combining a woven cotton scaffold, CI microparticles, and microwave-synthesized iron oxide microfibers into hybrid textile membranes that are directly integrated as dielectric layers in planar capacitors and then evaluated under coupled magnetic and mechanical stimuli.

In this work, we fabricate hybrid magneto-responsive textile membranes by impregnating woven cotton scaffolds with silicone oil (SO)-based suspensions containing CI microparticles and μFe. Three membranes with constant CI loading ($\Phi_{\mathrm{CI}}=10$ vol.%) and increasing μFe content (0, 10, and 20 vol.%) are integrated as dielectric layers in planar capacitors. Using capacitance measurements at 1 kHz under controlled magnetic flux density *B* and compressive pressure *p*, we estimate the apparent relative permittivity $\varepsilon_{r}$ and compare the magnetic-field sensitivity, pressure response, and apparent field-induced stiffening of the three membrane compositions. The obtained results are relevant for the design of flexible capacitor-based elements with coupled magnetic-field- and pressure-dependent response, including magnetic-field- and hydrostatic-pressure-sensing layers. More broadly, they may also be useful for the development of tunable capacitive elements whose response can be adjusted by magnetic field and mechanical deformation.

## 2. Materials and Methods

**Figure 1:**
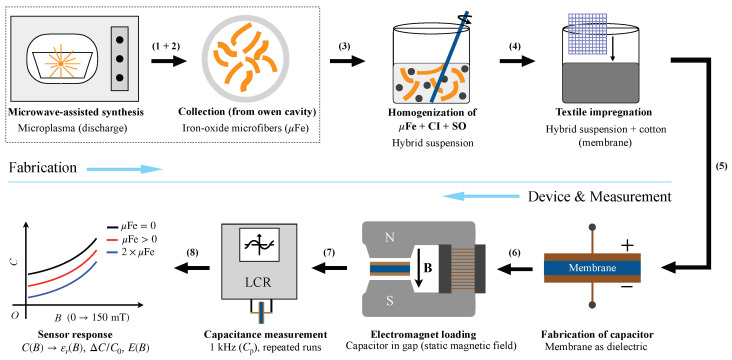
Schematic workflow for fabricating (**upper row**) and testing (**lower row**) hybrid CI/μFe cotton membranes. The two steps enclosed by the dashed frame (microwave-induced microplasma synthesis of μFe and their collection) were performed and reported previously in Ref. [14]. In this work, the μFe are co-dispersed with CI microparticles in SO to prepare hybrid suspensions, which are then used to impregnate cotton fabric and obtain flexible membranes. The membranes are further assembled as dielectric layers in planar capacitors, exposed to a static magnetic field, and characterized by capacitance measurements at 1 kHz. The capacitance was used to calculate $\varepsilon_{r}$, the normalized capacitance change, and the apparent modulus-like parameter *E*.

A schematic overview of the fabrication route (particle/fiber preparation, suspension formulation and textile impregnation), capacitor assembly, and magnetic-field-dependent electrical measurements, together with the main data reduction steps, is shown in Figure 1. Details for each step are provided in the following subsections.

### 2.1. Materials

- CI microparticles, purchased from Merck (Darmstadt, Germany) were used as received. According to the supplier, the CI microparticles have a mean diameter of about 5 µm (Appendix A) and a mass density $\rho_{\mathrm{CI}}=7.68$ g/cm^3^ at 24 °C. The specific saturation magnetization is $\sigma_{\mathrm{CI}}=195$ A·m^2^/kg, at magnetic field intensities H≳500 kA/m [24].
- The carrier liquid is SO, from Merck (Darmstadt, Germany), grade MS100, used as received (viscosity and density at 24 °C: 100 cSt and, respectively, $\rho_{\mathrm{SO}}=0.98$ g/cm^3^) [25]. SO was selected as the carrier liquid because it is electrically insulating, widely used in MRSs, and has a viscosity suitable for both dispersion of the magnetic phases and impregnation of the porous cotton scaffold.
- μFe were synthesized in-house, as described in Section 2.2 (see also Ref. [14]). Their average diameter is $d_{\mu \mathrm{Fe}}≃0.94$ µm and mass density $\rho_{\mu \mathrm{Fe}}=2.875$ g/cm^3^ at 24 °C. The specific saturation magnetization is $\sigma_{\mu \mathrm{Fe}}=22.7$ A·m^2^/kg, at H≳500 kA/m [14]. Supplementary morphological and elemental characterization is provided in Appendix B.
- Commercial cotton fabric (CF), from S. C. Universal Company S.A. (Salaj, Romania) was used as the porous textile scaffold. The fabric is a woven mesh of interwoven threads. The threads have a diameter $d_{t}\approx 0.12$ mm and are composed of cotton microfibers with a diameter $d_{y}\approx 5.5$ µm, leaving spaces that facilitate liquid uptake by capillarity [26]. Supplementary morphological and elemental characterization is provided in Appendix C.
- FR-4 laminate boards coated with copper (LAM75X100H, from ElectricTop (Bucharest, Romania), consisting of an epoxy resin/glass fiber reinforced substrate with a single-sided copper cladding. The laminate thickness is 0.6 mm and the board size is 100 mm × 75 mm. The nominal thickness of the copper plate is 18 µm.

### 2.2. Synthesis of μFe Microfibers

μFe microfibers were synthesized using a microwave-assisted microplasma route, following the protocol reported in [14]. Iron pentacarbonyl (Fe(CO)_5_) served as the iron precursor. A polyphasic precursor mixture consisting of CI microparticles, Fe(CO)_5_ and SO was homogenized in an open, heat-resistant glass beaker and exposed to microwave irradiation in a domestic microwave oven (2.45 GHz; Samsung MG23F301TAK, Suwon, Republic of Korea). The microwave output power and exposure time were set to 450 W and 120 s, respectively, as in Ref. [14]. The precursor composition was identical to Ref. [14] (CI: 2 cm^3^, Fe(CO)_5_: 1 cm^3^, SO: 5 cm^3^). After microwave treatment, a loose cotton-wool-like product composed of μFe was obtained [14]. After completion of the irradiation step, the product was cooled to room temperature and collected mechanically from the beaker and chamber surfaces. The microfibers collected were stored in sealed glass vials at room temperature until use in hybrid suspensions (Section 2.3). These processes correspond to the first two steps (1 + 2) enclosed by the dashed line, in Figure 1.

During microwave irradiation, a luminous microplasma and intermittent micro-explosion-like events can occur in the precursor mixture, promoting rapid decomposition of the iron precursor and the formation of transient metal vapor/gas streams. In cooler regions of the vessel and chamber, these vapors can condense (“dew-point” zones), generating ultrafine iron/iron oxide particulates that further undergo nonstationary mass transfer and aggregation into a fluffy, cotton-wool-like product. The resulting hierarchical microfiber network is therefore consistent with a vapor–condensation-assembly route occurring under microplasma-assisted microwave heating (see Ref. [14] for a detailed mechanistic discussion).

### 2.3. Preparation of Hybrid Suspensions

Hybrid MRSs, containing CI microparticles and μFe co-dispersed in SO, were prepared as described below. The volume of the components ($V_{\mathrm{CI}}$, $V_{\mathrm{SO}}$, $V_{\mu \mathrm{Fe}}$) was set to the values specified in Table 1. For each suspension, the corresponding volume fractions are calculated as follows:

(1) $$\Phi_{k}=\frac{V_{k}}{V_{\mathrm{CI}}+V_{\mathrm{SO}}+V_{\mu \mathrm{Fe}}},\;k\in \left\{\mathrm{CI},\;\mathrm{SO},\;\mu \mathrm{Fe}\right\}.$$

The suspensions were prepared following a common protocol:

Step 1: The volumes of CI microparticles, SO, and μFe microfibers were measured according to Table 1.
Step 2: The measured quantities were introduced into 20 mL Berzelius glass beakers (one beaker per composition).
Step 3: Each mixture was homogenized mechanically under heating (warm mixing) for approximately 300 s at about 150 °C.
Step 4: After the heating stage, thermal treatment was stopped, while mechanical homogenization was continued until the mixture temperature reached ambient temperature. At the end of this step, the MRSs were obtained (step (3) in Figure 1), with the volume fractions listed in Table 1.

During preparation, the temperature was monitored using an infrared thermometer (IRT-350 type, Conrad Electronic SE, Hirschau, Germany). The prepared suspensions were used for textile impregnation immediately after preparation.

### 2.4. Fabrication of Hybrid Cotton Membranes

Hybrid CI/μFe cotton membranes were manufactured by impregnating CF with freshly prepared hybrid MRS_1_–MRS_3_. Square CF specimens with a side length of 30 mm were cut from the same fabric roll. For each composition, a CF sample was placed in a 20 mL Berzelius glass beaker and impregnated with the corresponding suspension by adding an equal amount of aliquot (sufficient to fully wet/submerge the textile) and allowing capillarity uptake.

To promote infiltration into the textile network and improve the uniformity of the dispersion, each beaker containing the CF sample and suspension was subjected to a short warm treatment (approximately 300 s) while it was mechanically agitated. The beakers were then allowed to cool to ambient temperature. The impregnated samples were left to equilibrate at room temperature for 24 h. After this period, the resulting membranes were removed and placed above a Petri dish (or on an inert support) to allow removal of visible excess liquid by gravitational draining.

The membranes were labeled according to the suspension used for impregnation: hM_1_ (from MRS_1_), hM_2_ (from MRS_2_) and hM_3_ (from MRS_3_). The CI loading was kept constant in the MRSs at $\Phi_{\mathrm{CI}}=20$ vol.% (Table 1). After impregnation, the volume fractions of the membrane change because the cotton scaffold contributes an additional volume. Consequently, the volume fraction of CI in the membranes is $\Phi_{\mathrm{CI}}=10$ vol.% for all samples (Table 2). The membrane thickness measured after draining was approximately the same for all three compositions, namely d≈0.42 mm. The membranes were stored in sealed containers at room temperature until the capacitor assembly (Section 2.5).

**Figure 2:**
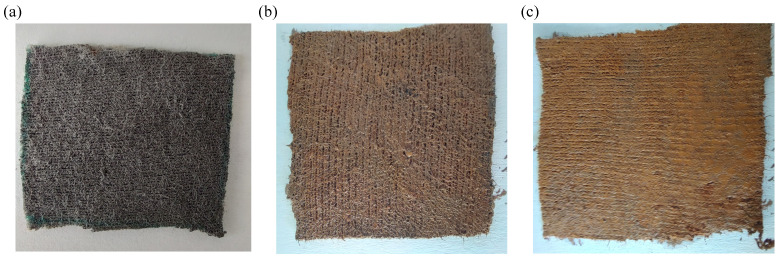
Photographs of the hybrid membranes prepared with increasing μFe loading. (**a**) hM_1_. (**b**) hM_2_. (**c**) hM_3_. The membrane color changes from black (hM_1_) to progressively more brownish tones at higher μFe content. The edge of each membrane is 30 mm.

The hybrid membranes (hM_*i*_, i=1,2,3) obtained by cotton impregnation (Figure 2) have the compositions summarized in Table 2. This corresponds to step (4) in Figure 1. For each membrane, the volumes of the components (CI, SO, CF scaffold and μFe) and the corresponding volume fractions are reported.

After impregnation, the volume fractions in the membranes were calculated analogously to Equation (1), but with the additional contribution of the cotton-fabric scaffold included in the total volume. Thus, the membrane fractions were determined from

(2) $$\Phi_{k}=\frac{V_{k}}{V_{\mathrm{CI}}+V_{\mathrm{SO}}+V_{\mu \mathrm{Fe}}+V_{\mathrm{CF}}},\;k\in \left\{\mathrm{CI},\mathrm{SO},\mu \mathrm{Fe},\mathrm{CF}\right\},$$

where $V_{\mathrm{CF}}$ is the volume of the cotton scaffold. Consequently, the volume fraction of CI in the membranes becomes $\Phi_{\mathrm{CI}}=10$ vol.% for all samples (Table 2).

### 2.5. Planar Capacitor Assembly

**Figure 3:**
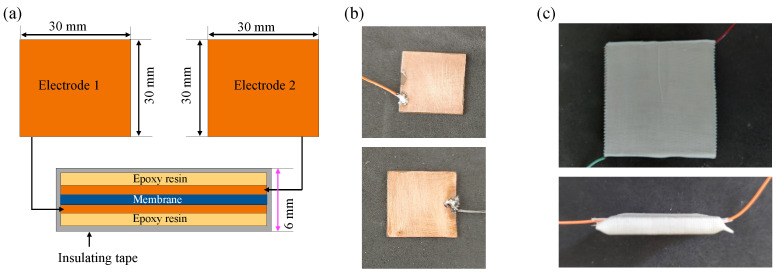
Planar capacitor design and assembly used for capacitance measurements. (**a**) Schematic of the device: top—plan view of the two copper electrodes; bottom—cross-sectional stack (not to scale) comprising FR-4/epoxy substrate–copper electrode–hybrid membrane–copper electrode–FR-4/epoxy substrate, enclosed and laterally aligned using insulating adhesive tape. (**b**) Photographs of the individual electrodes with attached electrical leads. (**c**) Photographs of the assembled capacitor: top—top view of the taped device with the two electrical connections; bottom—side/cross-sectional view showing the stacked configuration.

Hybrid membranes hM_*i*_ (i = 1, 2, 3) were used as dielectric layers in parallel capacitors PC_*i*_ (i = 1, 2, 3) manufactured from single-sided copper-clad FR-4 boards. The copper cladding served as the electrode material. For each capacitor, two identical electrodes were prepared and arranged face-to-face, with the membrane placed between them. The electrode overlap area *A* was defined by the geometry of the copper pad (Figure 3a) and was kept constant for all measurements. Here, it corresponded to a square region of 30mm×30mm. Then, each membrane was centered on the lower electrode so that the overlap area was fully covered by the membrane. The upper electrode was then aligned to ensure a reproducible overlap area and to minimize lateral misalignment. This corresponds to step (5) in Figure 1.

The capacitor stack (FR-4/copper electrode–membrane–copper electrode/FR-4) was laterally aligned and enclosed using an insulating adhesive tape (3M) to ensure reproducible positioning and mechanical integrity (Figure 3a). The tape helped maintain the alignment of the stack and provided a mild mechanical restraint. The electrical connection to the copper electrodes was made using insulated leads attached to the electrodes (Figure 3b).

The total thickness of the capacitor (including the supports and FR-4 tape) was approximately 6 mm; however, in the capacitance analysis, the effective separation of the electrode was taken as the membrane thickness *d* measured by a micrometer. Representative photos of the assembled device are shown in Figure 3c.

### 2.6. Magnetic-Field Setup and Electrical Measurements

**Figure 4:**
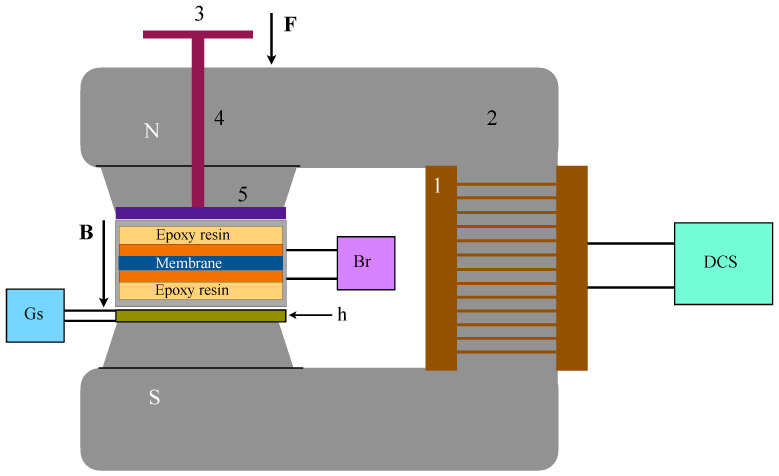
Schematic of the experimental setup used to apply a static magnetic field and a controlled normal load to the planar capacitor. The custom-built EM comprises the coil (1) and magnetic yoke (2), driven by a DCS power supply. The capacitor stack is positioned in the pole gap and connected to the LCR bridge (Br) for capacitance measurements. *B* in the sample region is monitored using a Hall probe h placed beneath the stack and read out by a gaussmeter (Gs). The assembly is mounted on a textolite support plate (3) and aligned using a brass rod (4, diameter 8 mm) and a brass plate (thickness 1.5 mm). Here, **F** is the force vector.

Capacitance measurements were performed with the planar capacitor (Section 2.5) placed in the air gap between the N and S poles of a custom-built electromagnet (EM). This corresponds to step (6) in Figure 1. EM consists of a coil (Figure 4, position 1) wound on a ferromagnetic yoke (Figure 4, position 2). The pole gap was set at 6 mm. The coil was powered in direct-current source (DCS) mode by a regulated power supply (RXN-3020D, Rexton Electronics Co., Ltd., Zaoxin, China), allowing continuous tuning of *B* up to ≈150 mT.

The *B* in the sample region was monitored using a gaussmeter (Dexing Magnet, Xiamen, China) equipped with a Hall probe (Figure 4, h). During measurements, the Hall probe was positioned beneath the capacitor stack, aligned with the center of the electrode overlap area, to provide a reproducible reading of the local magnetic field experienced by the sample.

Capacitance was measured using an inductance–capacitance–resistance (LCR) bridge (CHY-41R, Taiwan, China) connected to the capacitor electrodes (Figure 4, Br). This corresponds to step (7) in Figure 1. Measurements were performed at a fixed frequency of 1 kHz in a parallel equivalent-circuit configuration appropriate for a planar capacitor.

In addition to the magnetic field, a controlled normal load could be applied to the capacitor stack along the direction of the magnetic field (Figure 4). A vertical hole drilled through the yoke allows a loading rod to pass. Masses (m) are placed on the loading platform to generate a compressive force F=mg, corresponding to p=F/A, where *A* is the area of overlap of the electrode (Section 2.5). For each membrane, the capacitor was mounted in the EM gap using identical positioning and tapping conditions. The magnetic field was set by adjusting the DCS current supplied to the coil, and the corresponding value *B* was read from the gaussmeter. The curves C(B) and C(p) reported correspond to single representative measurement series. Succesive magnetic-field sweeps were not averaged since the capacitor response exhibits measurable hysteresis and memory effects under magnetic-field cycling (see Figure A4 in Appendix D).

From the measured field and load-dependent capacitance, we subsequently determined $\varepsilon_{r}$ of the membranes, the magnetic stress ($\tau_{\mathrm{zz}}$), the apparent strain ($e_{\mathrm{zz}}$), and the apparent modulus-like parameter (*E*) as described in Section 2.7. This corresponds to step (8) in Figure 1.

### 2.7. Data Reduction and Extracted Quantities

For each membrane (hM_*i*_, i=1,2,3), the equivalent capacitance C(B,p) of the planar capacitor (Section 2.5) was measured at f=1 kHz (Section 2.6) as a function of *B* and *p*.

To quantify the magnetic-field-induced capacitance change, we define the magnetocapacitance as

(3) $$\mathrm{MC}\left(B,p\right)=\frac{C(B,p)-C(0,p)}{C(0,p)}\times 100\%.$$

Here, C(B,p) is the capacitance measured at *B* and *p*, while C(0,p) is the corresponding zero-field capacitance measured at the same *p*.

To quantify pressure-sensing performance, we define the average normalized pressure sensitivity as

(4) $$S_{p}\left(B\right)=\frac{\left[C\left(B,p_{\max}\right)-C\left(B,0\right)\right]/C\left(B,0\right)}{p_{\max}},$$

where C(B,0) is the capacitance at p=0 kPa, $C(B,p_{\max})$ is the capacitance at maximum applied pressure $p_{\max}$, and $S_{p}\left(B\right)$ represents the average normalized pressure sensitivity over $0\leq p\leq p_{\max}$.

The quantities directly measured are the capacitances at the device-level C(B,p) of the assembled capacitor structures. The dielectric and mechanical-response quantities introduced in the following are model-derived effective or apparent parameters. Although the primary observables are C(B,p) of the assembled capacitor, the quantities reported below are attributed to the hybrid membrane within a simplified description of the effective medium. In this framework, the field- and load-dependent response is assumed to be dominated by the dielectric layer, while the electrodes and supports are treated as geometrically and electrically invariant. Consequently, $\varepsilon_{r}$ is used here as an apparent relative permittivity, $e_{\mathrm{zz}}$ as a capacitance-derived proxy, and *E* as an apparent modulus-like parameter derived within the adopted magnetic-stress model.

The adopted capacitance-based approach offers the advantage of probing the membrane response directly in the working capacitor configuration under combined magnetic and mechanical loading. Within this framework, the mechanical-response quantities discussed below are inferred indirectly from the capacitance data, and no separate independent mechanical tests (e.g., direct compression or tensile measurements) were carried out.

Using the parallel-plate capacitor relation, $\varepsilon_{r}$ of membrane *i* (i=1,2,3) at a given *B* was calculated using the zero-field thickness as

(5) $$\varepsilon_{r}\left(B\right)=\frac{C(B,0)\;d}{\varepsilon_{0}\;A},$$

where $\varepsilon_{0}$ is the vacuum permittivity, *A* is the electrode overlap area, and *d* is the membrane thickness. For the present capacitors, $A\equiv L^{2}$, L=30 mm (see Figure 3) and d=0.42 mm. Unless stated otherwise, $\varepsilon_{r}\left(B\right)$ is evaluated at p=0. Because the membrane thickness may change under magnetic field and the dielectric layer is structurally heterogeneous, $\varepsilon_{r}\left(B\right)$ obtained in this way is an apparent effective quantity.

Because the measured capacitance increases under a magnetic field, the field-induced deformation is assumed to be predominantly compressive. To obtain a comparative measure of deformation from capacitance data, we estimate [27]

(6) $$e_{\mathrm{zz}}=\frac{C(0,p)}{C(B,p)}-1,$$

where C(0,p) and C(B,p) are the capacitances measured at the same *p* in the absence and presence of a magnetic field, respectively.

Following the dipolar interaction approximation adopted in [28], the magnetic interaction force acting on the membrane can be approximated as

(7) $$F_{m}\approx -9\Phi_{\mathrm{CI}}L^{2}B^{2}/\left(2\mu_{0}\right),$$

from which we obtain:

(8) $$\tau_{\mathrm{zz}}=F_{m}/L^{2}\approx -9\Phi_{\mathrm{CI}}B^{2}/\left(2\mu_{0}\right).$$

In the following mechanical analysis, $\Phi_{\mathrm{CI}}$ refers to the volume fraction of CI microparticles in the final membrane (Table 2), not in the precursor suspension (Table 1). Therefore, for the membranes investigated here, $\Phi_{\mathrm{CI}}=10$ vol.% and $\mu_{0}=4\pi \times 10^{-7}$ H/m, so Equation (8) becomes

(9) $$\tau_{\mathrm{zz}}\left(\mathrm{Pa}\right)\approx -0.358\;B^{2}\left(\mathrm{mT}\right).$$

The negative sign indicates that the membranes undergo compression in the applied magnetic field.

An apparent modulus-like parameter of each membrane can then estimated from the ratio between $\tau_{\mathrm{zz}}$ and field-induced strain:

(10) $$E=\frac{\tau_{zz}}{e_{\mathrm{zz}}}.$$

Because $\tau_{\mathrm{zz}}$ is estimated from a simplified dipolar magnetic-stress model and $e_{\mathrm{zz}}$ is indirectly inferred from capacitance, *E* should be interpreted here as an apparent comparative stiffness indicator.

## 3. Results

### 3.1. Morphology, Composition, and Magnetic Properties of μFe

The morphology, composition, phase constitution, and magnetic properties of the μFe used here were previously established in [14]; only a brief summary is given because these characteristics define the secondary magnetic filler in the hybrid CI/μFe membranes. Scanning electron microscopy (SEM) observations showed that the microwave-synthesized microfibers form a complex hierarchical spiderweb-like network of interconnected filaments, with diameters in the range of approximately 0.25–2.20 µm and an average diameter of about 0.94 µm [14]. Energy-dispersive X-ray spectroscopy (EDX) analysis confirmed that the microfibers contain Fe and O, while X-ray diffraction (XRD) measurements revealed a mixed-phase iron oxide composition consisting of α-Fe_2_O_3_, γ-Fe_2_O_3_, and Fe_3_O_4_ [14].

Room-temperature magnetic measurements further showed that the microfibers are magnetically active, with a specific saturation magnetization of $\sigma_{\mu \mathrm{Fe}}\approx 22.7$ A·m^2^/kg, a remanent magnetization of approximately 2.86 A·m^2^/kg, and a coercive field of approximately 12.3 kA/m [14]. These values are substantially lower than those of CI microparticles (see Appendix A), which is consistent with the mixed-phase oxidized nature of the microfibers. At the same time, the hierarchical morphology and mixed iron oxide composition of the μFe provide structural and magnetic features that are complementary, rather than redundant, to those of CI. Therefore, when incorporated together with CI in cotton-based membranes, μFe are expected to act as a secondary magnetic filler that modifies the packing of particles, constrains the field-induced rearrangement and thereby helps tune the dielectric and apparent mechanical response of the hybrid CI/μFe textile membranes investigated here.

### 3.2. Membrane Morphology and Structure

**Figure 5:**
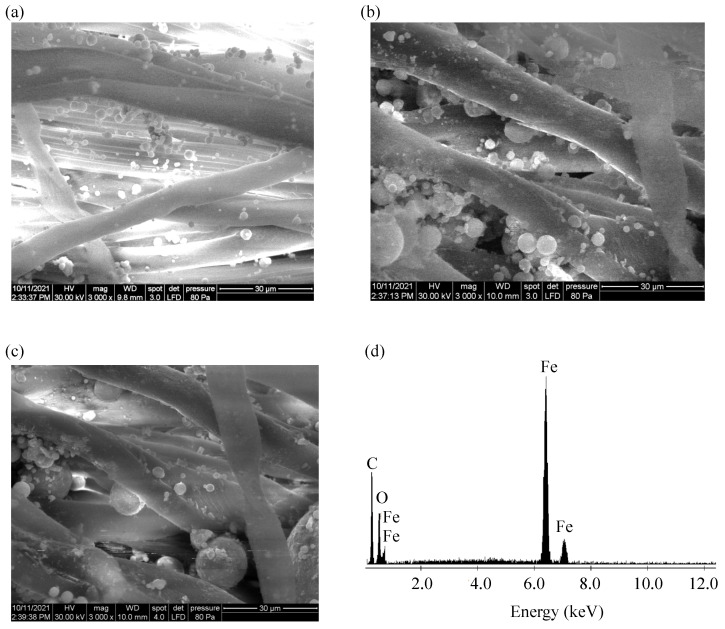
Representative morphological and elemental characterization of the hybrid CI/μFe membranes. SEM images of (**a**) hM_1_, (**b**) hM_2_, and (**c**) hM_3_ were acquired at the same magnification for direct comparison. (**d**) Representative EDX spectrum of the hybrid membranes. The EDX spectra of hM_1_, hM_2_, and hM_3_ were qualitatively similar; therefore, only one representative spectrum is shown for clarity. CI—large particles, μFe—small particles/fragments.

To study the morphology and structure of the hybrid membranes, a SEM Inspect S PANalytical microscope from FEI Company (Eindhoven, The Netherlands) was used in low-vacuum mode at an accelerating voltage of 30.0 kV, coupled with an EDX detector operating up to about 12 keV. Representative SEM images and EDX spectra of the fabricated hybrid CI/μFe membranes are shown in Figure 5.

**Figure A3:**
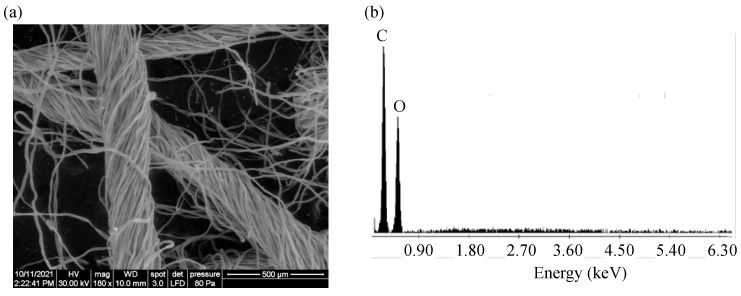
Supplementary morphological and elemental characterization of μFe microfibers used. (**a**) SEM image; (**b**) EDX spectrum, confirming the Fe/O composition of the microfibers.

After impregnation, the membranes preserve the overall fibrous texture of the cotton scaffold, but the SEM images reveal additional solid-phase features associated with the incorporated magnetic filler. Compared with pristine fabric (shown in Figure A3), the hybrid membranes shown in Figure 5 appear denser and less open, indicating that the magnetic solid phases are distributed within and around the cotton-fiber network. The iron oxide phase no longer appears exclusively as extended microfibers, but also as smaller oxide-rich fragments and particulate domains distributed both around the CI microparticles and in the free spaces between the cotton microfibers. This behavior is consistent with redistribution of the μFe phase during homogenization and textile impregnation. The corresponding EDX spectrum confirms the presence of Fe-containing inorganic material in the membrane, demonstrating successful incorporation of the magnetic phase into the textile structure. Therefore, SEM/EDX observations show that the hybrid membranes retain the flexible fibrous framework of the cotton substrate while accommodating both large CI microparticles, together with smaller iron-oxide-rich particles, within the inter-fiber voids.

### 3.3. Capacitance

**Figure 6:**
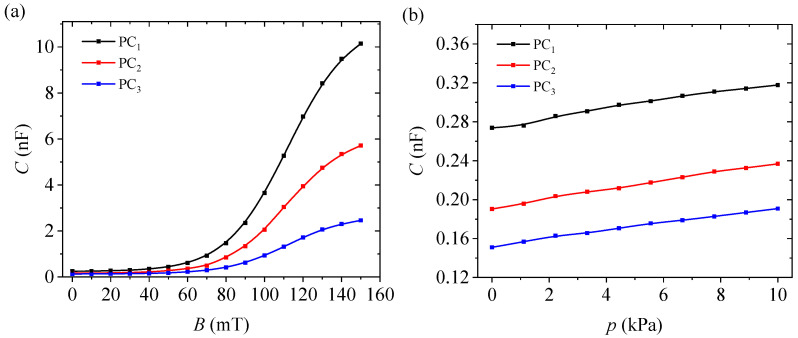
Capacitance response of the three planar capacitors based on hybrid CI/μFe cotton membranes under magnetic and mechanical stimuli. (**a**) Capacitance, *C*, as a function of *B*, measured at p=0 kPa. (**b**) Capacitance, *C*, as a function of *p*, measured at B=0 mT. Points-experimental data. Continuous lines—interpolation.

**Figure A4:**
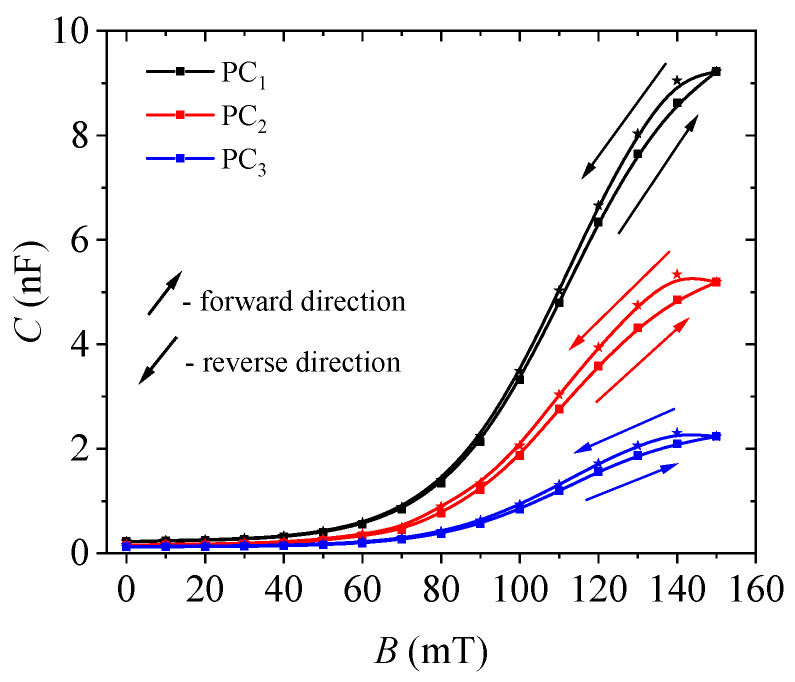
Hysteresis of the capacitance response as a function of *B* for the three planar capacitors PC_1_, PC_2_, and PC_3_. The magnitude of the hysteresis is highest for PC_1_, intermediate for PC_2_, and lowest for PC_3_. Points—experimental data. Continuous lines—interpolation.

The performance of membrane-based capacitors fabricated as flexible capacitive elements was first assessed by direct electrical measurements under magnetic and mechanical stimuli. For this purpose, the capacitance of the three planar capacitors PC_1_–PC_3_ was recorded as a function of *B* at p=0 kPa (Figure 6a), as a function of *p* at B=0 mT (Figure 6b), and during magnetic field cycling to examine hysteresis effects (Figure A4 in Appendix D).

The capacitance of all three planar capacitors increased monotonically with *B* in the investigated range 0–150 mT. In zero field, the capacitance values were 0.259 nF, 0.182 nF, and 0.145 nF for PC_1_, PC_2_, and PC_3_, respectively, and this ordering was preserved throughout the magnetic-field range. The increase was weak at low magnetic flux densities and became much more pronounced above about 60–80 mT, indicating a strongly nonlinear magnetic response. At B=150 mT, the capacitance reached 9.255 nF for PC_1_, 5.222 nF for PC_2_, and 2.261 nF for PC_3_, corresponding to increases of approximately 35.7, 28.7, and 15.6 times relative to their zero-field values.

A monotonic increase in capacitance was also observed with *p*. In the investigated interval 0–10 kPa, the capacitance is quasi-linear for all three capacitors. It increased from 0.259 to 0.301 nF for PC_1_, from 0.182 to 0.227 nF for PC_2_, and from 0.145 to 0.176 nF for PC_3_. These changes correspond to relative increases of approximately 16.2%, 24.7%, and 21.4%, respectively. Although PC_1_ retained the highest absolute capacitance throughout the pressure range, PC_2_ displayed the largest absolute and relative pressure-induced increase. The data show that the three capacitor structures are sensitive to both magnetic field and compressive-pressure stimuli, with a much stronger absolute response to the magnetic field than to mechanical loading in the tested ranges.

For a compact comparison of the sensing characteristics, the main metrics extracted from C(B) and C(p) are summarized in Table 3.

### 3.4. Magnetic Field Dependences of Apparent Relative Permittivity and Apparent Strain

**Figure 7:**
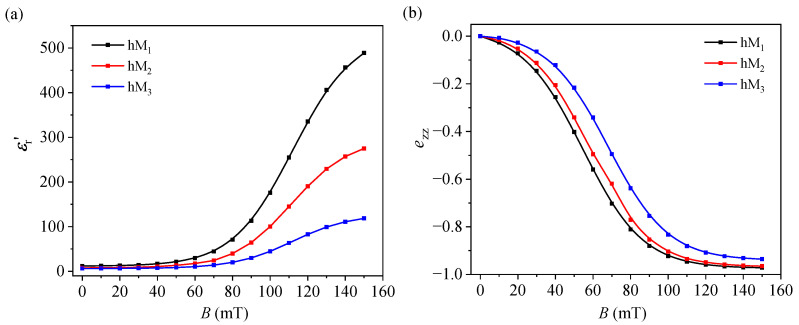
Magnetic field dependences of (**a**) $\varepsilon_{r}$, calculated with Equation (5), and (**b**) $e_{\mathrm{zz}}$, calculated with Equation (6), for membranes hM_1_, hM_2_, and hM_3_.

The capacitance data were further used to evaluate $\varepsilon_{r}$, and the capacitance-derived proxy $e_{\mathrm{zz}}$, as functions of *B*. The corresponding results were calculated using Equations (5) and (6) and are presented in Figure 7. $\varepsilon_{r}$ increases monotonically with the *B* for all three membranes, while the $e_{\mathrm{zz}}$ component becomes progressively more negative. In both cases, the magnitude of the response decreases in the order hM_1_ > hM_2_ > hM_3_. For low magnetic fields, the variations of $\varepsilon_{r}$ and $e_{\mathrm{zz}}$ are relatively weak, whereas much stronger changes occur in the intermediate- and high-field range, followed by a tendency toward saturation at the highest values of *B*. At B=150 mT, $\varepsilon_{r}$ reaches approximately 488.8, 275.0, and 118.5 for hM_1_, hM_2_, and hM_3_, respectively, while $e_{\mathrm{zz}}\approx -0.972$, −0.965, and −0.936.

### 3.5. Magnetic Stress and Apparent Modulus-like Response

**Figure 8:**
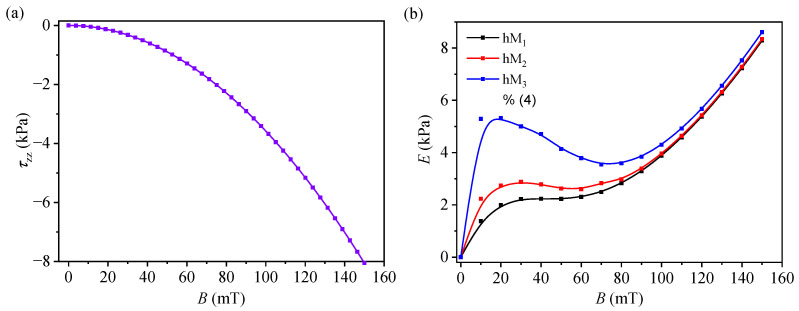
Magnetic-field dependences of (**a**) $\tau_{zz}$, calculated from Equation (9), and (**b**) the apparent modulus-like parameter, *E* (kPa), for membranes hM_1_, hM_2_, and hM_3_, calculated from Equation (10).

$\tau_{zz}$ calculated from Equation (9) and the corresponding apparent modulus-like parameter *E* (kPa) are shown in Figure 8. As expected from Equation (9), the normal $\tau_{\mathrm{zz}}$ is compressive and becomes increasingly negative with increasing *B*, changing from $\tau_{zz}=0$ at B=0 to $\tau_{zz}\approx -8.06$ kPa at B=150 mT. The magnitude of $\tau_{zz}$ increases nonlinearly throughout the investigated field range, reflecting its approximately quadratic dependence on *B*.

The apparent modulus-like parameter exhibits a membrane-dependent behavior at low and intermediate magnetic fields, followed by a clear increase at higher fields for all three membranes. At B=10 mT, the apparent modulus values are approximately 1.38, 2.23, and 5.29 for hM_1_, hM_2_, and hM_3_, respectively, indicating that hM_3_ is the most rigid membrane in the low-field range. For hM_1_, *E* gradually increases from approximately 1.38 at 10 mT to 8.29 at 150 mT. For hM_2_, *E* increases from about 2.23 at 10 mT to 8.35 at 150 mT, with a weak minimum at around 50–60 mT. For hM_3_, the modulus is initially the highest, decreases from about 5.32 at 20 mT to about 3.54 at 70 mT, and then increases again to about 8.61 at 150 mT. At the highest applied field, the apparent modulus values are close for all three membranes, in the order hM_3_ > hM_2_ > hM_1_.

For a compact sensor-oriented comparison, the selected dielectric and apparent mechanical metrics derived from the membranes hM_1_, hM_2_, and hM_3_ are summarized in Table 4. The values show that hM_1_ has the highest $\varepsilon_{r}$ and magnetocapacitance, while hM_3_ exhibits the lowest dielectric response and the highest apparent modulus-like parameter in the intermediate magnetic field. $e_{\mathrm{zz}}$ at B=150 mT remains strongly negative for all three membranes, while the apparent stiffening ratio decreases from hM_1_ to hM_3_.

## 4. Discussion

### 4.1. Origin of the Magnetic-Field-Induced Dielectric Response

Both *C* and $\varepsilon_{r}$ increase monotonically with *B* for all membranes (Figure 6a and Figure 7a). In the present planar capacitor configuration, this behavior is consistent with the magnetic-field-induced restructuring of the CI-rich phase and the resulting changes in the electrical response of the heterogeneous dielectric layer [28,29]. Under an applied magnetic field, the CI microparticles become magnetized and tend to interact along the field direction, which can promote the development of field-induced structures within the cotton scaffold impregnated with SO [29,30,31]. In this framework, the magnetocapacitive response can be attributed to field-induced reorganization of the CI-rich phase into chain-like or columnar structures [28,29,30]. This reorganization modifies interfacial polarization, effective permittivity, and polarization/charge-transport pathways across the heterogeneous dielectric layer.

The response is distinctly nonlinear. At a low field, the capacitance changes only slightly and then rises much more rapidly in the intermediate range, before approaching saturation at the highest fields. This progression is consistent with the field-induced structuring within the membrane [29]. At low *B*, only limited rearrangement is expected, while at higher *B* the magnetic interactions become strong enough to induce a much larger change in the internal configuration of the membrane. The smaller incremental variation observed at the highest fields suggests that the system approaches a near-saturated state, so that further increases in *B* produce only modest dielectric changes. Thus, the nonlinear shape of the C(B) and $\varepsilon_{r}\left(B\right)$ curves is consistent with progressive field-induced formation and densification of CI-rich structures, together with the resulting changes in interfacial polarization and effective dielectric pathways; at higher fields, the response tends toward saturation [28,29,30].

The simultaneous evolution of $\varepsilon_{r}$ and $e_{zz}$ further indicates that the dielectric response is coupled to the apparent compressive deformation. As the magnetic field increases, $\varepsilon_{r}$ increases, while $e_{zz}$ becomes increasingly negative. This indicates that the dielectric enhancement is linked both to changes in the polarization pathways and to field-induced changes in the internal geometry of the composite layer. In this sense, the capacitance increase under magnetic field is interpreted here not as a purely dielectric effect in a fixed geometry, but as the coupled result of dielectric pathway reorganization and apparent field-induced compaction of the soft composite layer. The negative evolution of $e_{zz}$ is consistent with this interpretation, since magnetic attraction between neighboring CI-rich domains tends to compress the membrane along the field direction, and thereby contributes to the observed increase in capacitance [27,28].

### 4.2. Effect of μFe Loading on Magnetocapacitance and Apparent Deformation

As the fraction of μFe increases, the zero-field capacitance decreases, the magnetic-field-induced capacitance change decreases, and $\varepsilon_{r}$ in a given field is reduced (Figure 6a and Figure 7a). The same trend is observed for the magnitude of $e_{\mathrm{zz}}$: at B=150 mT, the absolute value of $e_{zz}$ is highest for hM_1_ and lowest for hM_3_ (Figure 7b). Therefore, increasing the μFe content reduces both the magnetocapacitance and the magnitude of the apparent deformation derived from the capacitance.

A simple way to interpret these results is to treat the CI-rich phase in the porous textile as a chain-like structure embedded in an oxide-/polymer-/oil-rich dielectric background. In such a microstructural picture, the device-level capacitance reflects the combined contribution of many local CI–dielectric–CI interfaces arranged in series and parallel within the electrode overlap area. Increasing the fraction of μFe introduces an additional oxide-rich phase that modifies the spatial arrangement of the CI-rich domains and increases the contribution of weakly polarizable regions to the effective dielectric pathway. Both effects can lower the zero-field capacitance and alter the field-induced reorganization of the CI-rich phase, thereby reducing the overall magnetocapacitive response. The added fibrous phase may also mechanically constrain the rearrangement of the CI-rich domains, consistent with the observed increase in apparent low-field stiffness at higher μFe loading. Thus, the role of the μFe component may be viewed primarily as microstructural modification rather than as the main magnetic phase in comparison with CI. The additional oxide-rich phase increases the characteristic spacing between CI-rich regions and modifies the local geometry of the interparticle dielectric gaps. As a result, the zero-field capacitance is reduced, and the subsequent field-induced dielectric response is altered [17].

This behavior is consistent with the different roles played by the two magnetic components. CI remains the dominant magnetically active phase in the membranes because its saturation magnetization is much higher than that of the μFe (Section 2.1; Ref. [14]). In contrast, μFe, due to their lower magnetization and fibrillar morphology, appear to act mainly as a secondary structural component that modifies the internal spatial arrangement of the composite. A higher μFe loading would then be expected to modify or hinder the field-induced chain-like structure of the CI-rich phase and thereby alter the field-responsive performance, while also improving dispersion stability through steric/structural effects [32]. This interpretation is also consistent with the observed reduction in the magnitude of $e_{\mathrm{zz}}$ as the μFe loading increases. If the oxide-rich phase partly restricts the approach and compaction of neighboring CI-rich domains under magnetic field, then both the apparent compressive deformation and the associated capacitance increase should become smaller, as observed experimentally. In this sense, the μFe phase appears to contribute not only to the dielectric background, but also to the mechanical confinement of the field-induced CI restructuring within the membrane [14,28,29,32].

The SEM observations are qualitatively consistent with this view. After impregnation, the cotton scaffold retains its fibrous character, but the membrane becomes denser and contains Fe-rich solid material distributed throughout the textile network (Figure 5). The revised SEM analysis further suggests that the solid phase is distributed within the inter-fiber voids of the cotton scaffold as a combination of large CI microparticles and smaller iron-oxide-rich domains or fragments. Within such a confined porous framework, the magnetic response may therefore be interpreted in terms of the combined action of CI dipolar interactions, μFe-mediated structural modification, and geometric/mechanical constraint imposed by the textile scaffold [17,28,32]. This combined picture explains why increasing the μFe content progressively suppresses the magnetocapacitive response while shifting the membrane behavior toward a more constrained apparent deformation.

### 4.3. Apparent Mechanical Response and Field-Induced Stiffening

The apparent modulus-like parameter *E* derived increases overall with *B* for all three membranes, although the detailed behavior in the low and intermediate fields depends on the composition (Figure 8b). In particular, hM_3_ exhibits the highest apparent modulus at low *B*, whereas hM_1_ is the softest. At higher fields, the modulus values of the three membranes become closer and all samples show a pronounced stiffening tendency. As discussed above, this apparent mechanical response is best interpreted together with the magnetocapacitive response, since both quantities are linked to the same field-induced restructuring process occurring within a mechanically constrained textile composite. Thus, the evolution of E(B) reflects not only the magnetic interactions between CI-rich domains, but also the extent to which the resulting restructuring is accommodated or hindered by the surrounding cotton scaffold and by the additional μFe-rich phase [28,31,33].

A useful interpretation is that, at low to intermediate *B*, the magnetic attraction between CI-derived dipoles is partially balanced by the elastic constraint of the cotton-fiber scaffold (and, at higher μFe loading, by the additional fibrous oxide network). In this regime, the membrane can exhibit a weak plateau or non-monotonic evolution of the apparent modulus because further dipole approach and chain compaction are mechanically constrained. At higher fields, dipolar interactions become strong enough to overcome these constraints, enabling denser field-aligned structures and producing the observed high-field stiffening. This means that the magnetic field affects not only the dielectric response but also the apparent resistance of the membranes to further compression. This behavior is consistent with bulk magneto-mechanical, magnetic-field-induced stiffening, which increases resistance to deformation as magnetic interactions grow [33]. The higher initial modulus of hM_3_ further suggests that increasing μFe loading stiffens the membrane, even before strong field-induced structuring is established. In this picture, the higher low-field values of *E* for the μFe-rich membranes are consistent with a more constrained internal architecture, in which the oxide-rich phase and the textile framework oppose the early-stage compaction of CI-rich domains. As the magnetic field increases, however, dipolar attraction is expected to become progressively more dominant, so that the three compositions evolve toward a similarly stiffened apparent state despite their different initial levels of constraint [31,33].

At the same time, the non-monotonic behavior observed for hM_2_ and especially for hM_3_ in the low-to-intermediate field range indicates that the apparent mechanical response is more complex than a simple monotonic stiffening law. This feature may reflect competition among magnetic interactions, the pre-existing constraints imposed by the cotton scaffold, and the additional constraint introduced by the μFe phase. It is therefore reasonable to interpret the apparent modulus-like parameter not as a direct elastic modulus, but as a comparative indicator of how the field-induced CI restructuring couples to the mechanically confined geometry of the membrane. Within this framework, the observed trends suggest that increasing the μFe content shifts the response toward a more constrained and less freely compactable microstructure, while still allowing pronounced magnetic-field-induced stiffening at sufficiently high *B*.

### 4.4. Validity and Limitations

$\varepsilon_{r}$ is an apparent quantity obtained for a heterogeneous dielectric layer composed of cotton fibers, silicone oil, CI microparticles, and μFe microfibers [34]. Therefore, it represents an effective device-level response rather than the intrinsic permittivity of a uniform material. $e_{\mathrm{zz}}$ is inferred from the capacitance variation under the assumption that the main geometric change occurs along the thickness direction and that the electrode area remains unchanged. In addition, $\tau_{\mathrm{zz}}$ is estimated from a simplified dipolar model that does not explicitly account for the detailed morphology of the textile scaffold or the μFe network. Consequently, the *E* obtained from Equation (10) describes the relative evolution of the membrane response under a magnetic field within the framework used here.

The electrical characterization reported here was performed at a single frequency (1 kHz). Thus, the present results should be viewed as an operating-point characterization of the fabricated capacitor structures, rather than as a full frequency-dependent dielectric analysis.

### 4.5. Implications for Flexible Capacitive Elements

From an application perspective, the membrane response can be tuned through composition. The preferred composition depends on whether magnetic sensitivity or mechanical constraint is the main requirement. The membrane without added μFe, hM_1_, exhibits the largest magnetic-field-induced capacitance change, the highest $\varepsilon_{r}$ and the largest magnetocapacitance. This makes it the most favorable composition when a strong magnetic-field-dependent electrical response is the main objective.

Increasing the loading of μFe reduces the magnetocapacitive response but increases the apparent stiffness of the membrane, particularly in the low-field range. In this sense, hM_3_ is less dielectrically responsive but exhibits a more mechanically constrained apparent response under the magnetic field. The intermediate composition hM_2_ provides a compromise between these two tendencies. Thus, the three investigated membranes define a practical trade-off: a lower μFe loading favors a stronger dielectric response, whereas a higher μFe loading favors higher apparent rigidity and lower magnetic-field-induced reconfiguration.

From an application perspective, the present membranes can be considered as dielectric layers for flexible capacitor-based elements whose response can be tuned through composition. Membranes with lower μFe loading are better suited for situations where a stronger magnetic-field-induced capacitance change is required, whereas membranes with higher μFe loading are more appropriate when a more mechanically constrained response is preferred. Their combined sensitivity to magnetic field and compressive loading also points to potential use in soft multifunctional capacitive components, for example in flexible sensing elements operating under both magnetic and mechanical stimuli. In this sense, the main practical significance of the present work lies not in a single specific device architecture, but in demonstrating a composition-dependent route for tuning the balance between magnetic responsiveness and mechanical constraint in textile-based capacitor structures.

The pressure-response data (Figure 6b) further indicate that the electrical behavior is not governed by stimulation of the magnetic-field alone. Although all three capacitors show a measurable increase in capacitance under compressive loading, the composition dependence differs from that observed under a magnetic field: PC_2_ provides the highest average pressure sensitivity in the investigated range, while PC_1_ provides the strongest magnetic-field-induced capacitance change. This suggests that the mechanisms controlling pressure-induced and magnetic-field-induced capacitance variations are related but not identical, and that the membrane composition can be selected according to the dominant external stimulus. In this sense, the present hybrid membranes are best viewed as composition-tunable dielectric layers for flexible capacitor-based elements with coupled magnetic-field- and pressure-dependent response.

## 5. Conclusions

Flexible hybrid CI/μFe cotton membranes were prepared by impregnating a woven cotton scaffold with SO-based suspensions containing CI microparticles and μFe. The resulting membranes were then used as dielectric layers in planar capacitors. The capacitor structures responded to both the magnetic field and compressive pressure (Figure 6). Over the investigated range of 0–150 mT, the capacitance increased monotonically for all three compositions, with the strongest magnetic-field-induced response observed for PC_1_ and the weakest for PC_3_. Under compressive loading in the range of 0–10 kPa, the capacitance also increased for all samples, while PC_2_ showed the highest average pressure sensitivity in the tested interval.

Within the simplified analysis adopted here, the capacitance data were also used to estimate several apparent dielectric and mechanical-response parameters, including apparent relative permittivity, a capacitance-derived strain proxy, magnetic stress, and an apparent modulus-like parameter. The apparent relative permittivity increased strongly with *B* while the capacitance-derived strain became increasingly negative. The same analysis indicated an overall increase in the apparent stiffness parameter with magnetic field. Therefore, the data indicate that increasing the μFe content suppresses the magnetically induced capacitance change and reduces the magnitude of the apparent deformation, while shifting the response toward higher apparent stiffness, especially at low magnetic fields.

In general, hybrid CI/μFe textile membranes behave as composition-tunable dielectric layers in flexible capacitor structures that respond to both magnetic field and compressive loading. The lower μFe loading favors a stronger magnetic-field-induced capacitance change, whereas the higher μFe loading is associated with a more mechanically constrained apparent response. This behavior makes such soft magnetic textile composites promising candidates for multifunctional capacitor-based sensing elements. Further work should address the frequency dependence, cycling stability, reversibility, and direct mechanical validation of the capacitance-derived apparent quantities.

## Figures and Tables

**Table 1:** Component volumes and volume fractions of CI, SO, and μFe used to prepare the hybrid MRSs.

	$V_{\mathrm{CI}}$ (cm^3^)	$V_{\mathrm{SO}}$ (cm^3^)	$V_{\mu \mathrm{Fe}}$ (cm^3^)	$\Phi_{\mathrm{CI}}$ (%vol.)	$\Phi_{\mathrm{SO}}$ (%vol.)	$\Phi_{\mu \mathrm{Fe}}$ (%vol.)
MRS_1_	0.800	3.200	0.000	20	80	0
MRS_2_	0.800	2.800	0.400	20	70	10
MRS_3_	0.800	2.400	0.800	20	60	20

**Table 2:** Component volumes and volume fractions of CI, SO, CF, and μFe in the hybrid membranes hM_*i*_ (i = 1, 2, 3).

	$V_{\mathrm{CI}}$ (cm^3^)	$V_{\mathrm{SO}}$ (cm^3^)	$V_{\mu \mathrm{Fe}}$ (cm^3^)	$V_{\mathrm{CF}}$ (cm^3^)	$\Phi_{\mathrm{CI}}$ (%vol.)	$\Phi_{\mathrm{SO}}$ (%vol.)	$\Phi_{\mu \mathrm{Fe}}$ (%vol.)	$\Phi_{\mathrm{CF}}$ (%vol.)
hM_1_	0.072	0.288	0.000	0.360	10	40	0	50
hM_2_	0.072	0.216	0.072	0.360	10	30	10	50
hM_3_	0.072	0.144	0.144	0.360	10	20	20	50

**Table 3:** Selected sensor-related metrics of the three planar capacitors, extracted from the capacitance dependences C(B) and C(p). Here, $C_{0}$ is the capacitance at B=0 and p=0, $C_{150}$ is the capacitance at B=150 mT and p=0, and $C_{10}$ is the capacitance at p=10 kPa and B=0. The magnetocapacitance at the maximum magnetic field, MC(150mT,0), was calculated using Equation (3). The quantity $\Delta C_{p}=C_{10}-C_{0}$ denotes the absolute capacitance change induced by *p*, while the pressure sensitivity $S_{p}\left(0\right)$ was calculated using Equation (4).

Sample	$C_{0}$	Magnetic Field Response		Pressure Response		
	**(nF)**	$C_{150}$ **(nF)**	MC **(150 mT, 0) (%)**	$C_{10}$ **(nF)**	$\Delta C_{p}$ **(nF)**	$S_{p}$ **(0) (kPa^−1^)**
PC_1_	0.259	9.255	3473.4	0.301	0.042	0.0162
PC_2_	0.182	5.222	2769.2	0.227	0.045	0.0247
PC_3_	0.145	2.261	1459.3	0.176	0.031	0.0214

**Table 4:** Representative derived apparent dielectric and mechanical-response metrics of membranes hM_1_, hM_2_, and hM_3_ under magnetic field. Here, $\varepsilon_{r,0}$ is the apparent relative permittivity at B=0, $\varepsilon_{r}\left(150\right)$ and $e_{\mathrm{zz}}\left(150\right)$ are the corresponding values at B=150 mT, and $R_{E}=E\left(150\right)/E\left(50\right)$ is the apparent stiffening ratio. Here, *E* is reported in kPa.

Membrane	$\varepsilon_{r,0}$	$\varepsilon_{r}$ (150)	$e_{\mathrm{zz}}$ (150)	E (50)	E (150)	$R_{E}$
hM_1_	13.65	488.796	−0.9720	2.2272	8.2869	3.72
hM_2_	9.59	275.035	−0.9652	2.6279	8.3459	3.18
hM_3_	7.64	118.529	−0.9359	4.1394	8.6070	2.08

**Figure A1:**
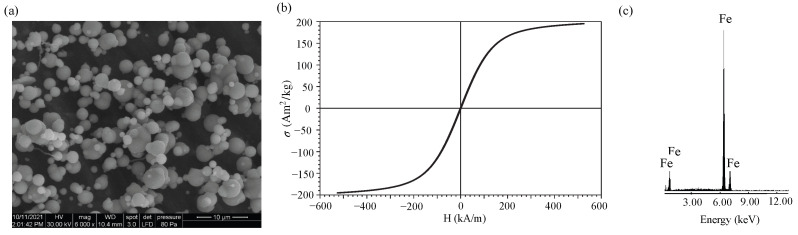
Supplementary characterization of the CI microparticles used in the present work. (**a**) SEM image. (**b**) Magnetization curve (obtained with an experimental setup described in Ref. [35]). (**c**) EDX spectrum.

**Figure A2:**
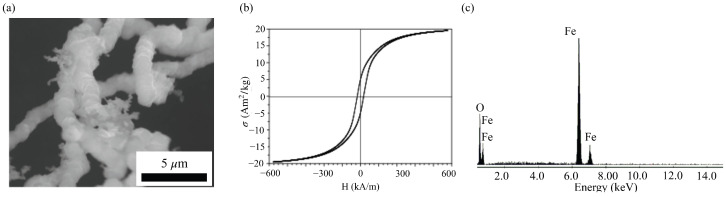
Supplementary morphological and elemental characterization of μFe microfibers used. (**a**) SEM image; (**b**) magnetization curve (obtained with an experimental setup described in Ref. [35]). (**c**) EDX spectrum, confirming the Fe/O composition of the microfibers.

## Data Availability

The data supporting the findings of this study are available within the article.

## Appendix A. Morphological and Magnetic Characterization of CI Microparticles

SEM image of CI microparticles confirms the nearly spherical morphology, with a mean diameter of approximately 5 µm (Figure A1a), while the magnetization curve (Figure A1b) shows a specific magnetization of approximately 195 A·m^2^/kg for H≈600 kA/m. (Figure A1c) shows the EDX spectrum of the CI, in which only Fe peaks are observed.

## Appendix B. Morphological and Elemental Characterization of Iron Oxide Microfibers

Figure A2 shows supplementary SEM, magnetization and EDX data for μFe microfibers used in this study. The SEM image (a) confirms the characteristic fibrous morphology of the microwave-synthesized material, and the magnetization curves (b) show that saturation magnetization is about 20 A·m^2^/kg for H≈600 kA/m. The substantially larger saturation magnetization of CI, compared with that of the μFe microfibers, supports its role as the primary magnetic filler in the hybrid membranes. Finally, the EDX spectrum (c) verifies that the microfibers contain Fe- and O-containing phases.

## Appendix C. Morphological and Elemental Characterization of Cotton Fabrics

Figure A3 shows supplementary SEM and EDX data for cotton used in this study. The pristine cotton fabric exhibits the expected interconnected fibrous architecture, with individual microfibers forming an open scaffold that contains voids suitable for suspension uptake during membrane fabrication. This morphology is consistent with the role of the textile as a flexible porous support for the magnetic filler dispersed in silicone oil. The EDX spectrum of the pristine cotton scaffold shows mainly C and O signals, consistent with the cellulosic composition of the fabric and the absence of the Fe-containing magnetic filler.

## Appendix D. Hysteresis Under Magnetic-Field Cycling

Figure A4 shows the hysteretic dependence of the capacitance on *B* for the three planar capacitors PC_1_–PC_3_. In all cases, the capacitance increases monotonically with increasing *B*, while during the reverse sweep the capacitance remains higher than on the forward branch at the same value of *B*. This behavior demonstrates a clear hysteretic magnetocapacitive response and indicates that the field-induced internal microstructure formed inside the hybrid CI/μFe membranes is only partially reversible when the magnetic field is reduced.

The hysteresis effect is most pronounced for PC_1_, decreases for PC_2_, and is weakest for PC_3_, which is consistent with the corresponding reduction in the overall magnetic-field-induced capacitance variation. Overall, these results show that the capacitor response is not only magnetically tunable, but also exhibits a measurable memory effect associated with the progressive formation and partial persistence of the internal field-induced structure.
